# The politics of the basic benefit package health reforms in Tajikistan

**DOI:** 10.1186/s41256-019-0104-4

**Published:** 2019-05-23

**Authors:** Eelco Jacobs

**Affiliations:** 1 University of Basel, Basel, Switzerland; 2 KIT Royal Tropical Institute, Amsterdam, The Netherlands

**Keywords:** Health governance, Health financing, Health policy, Health reform, Tajikistan, Political economy

## Abstract

**Background:**

Health reform is a fundamentally political process. Yet, evidence on the interplay between domestic politics, international aid and the technical dimensions of health systems, particularly in the former Soviet Union and Central Asia, remains limited. Little regard has been given to the political dimensions of Tajikistan’s Basic Benefit Package (BBP) reforms that regulate entitlements to a guaranteed set of healthcare services while introducing co-payments. The objective of this paper is therefore to explore the governance constraints to the introduction and implementation of the BBP and associated health management changes.

**Methods:**

This qualitative study draws on literature review and key informant interviews. Data analysis was guided by a political economy framework exploring the interplay between structural and institutional features on the one hand and agency dynamics on the other. Building on that the article presents the main themes that emerged on structure-agency dynamics, forming the key governance constraints to the BBP reform and implementation.

**Results:**

Policy incoherence, parallel and competing central government mandates, and regulatory fragmentation, have emerged as dominant drivers of most other constraints to effective design and implementation of the BBP and associated health reforms in Tajikistan: overcharging and informal payments, a weak link between budgeting and policymaking, a practice of non-transparent budget bargaining instead of a rationalisation of health expenditure, little donor harmonisation, and weak accountability to citizens.

**Conclusion:**

This study suggests that policy incoherence and regulatory fragmentation can be linked to the neo-patrimonial character of the regime and donor behaviour, with detrimental consequences for the health system.. These findings raise questions on the unintended effects of non-harmonised piloting of health reforms, and the interaction of health financing and management interventions with entrenched power relations. Ultimately these insights serve to underline the relevance of contextualising health programmes and addressing policy incoherence with long horizon planning as a priority.

## Background

Over the past fifteen years, reform processes in the health sector have been launched in Tajikistan to overhaul the inherited Semashko^1^ health system and address the high level of out-of-pocket payments on health. Among these reforms is the introduction of the Basic Benefit Package (BBP). The BBP, the first pilots of which started in 2004–2005, regulates entitlements to a specific, guaranteed set of healthcare services through a set of rules with pre-determined levels of co-payment charges and exemptions for categories of the population and patients. Supported by a constitutional amendment removing the right to free healthcare, the BBP reforms allow for an increase in revenues for the health system by formalizing informal payments and inverts the health system service delivery pattern relying heavily on the hospital level by redirecting resources to primary health care (PHC). However, its implementation has remained challenging.

Many of the policy details and organizational flaws of the health reforms in Tajikistan have been discussed from a variety of approaches [1–5]. The literature has exposed the main technical weaknesses of the system and its symptoms, including oversupply of specialised care at the expense of PHC; inefficient budget formulas; weak information systems; and its outcomes in the areas of epidemiology, quality of and access to health care. External assistance has come into the country to address many of these challenges in a slow process of moving from relief to development aid in the first decade after the end of the civil war [6]. However, despite progress in some areas there has been insufficient consideration of the fact that“ long-term results are contingent upon the murkier, less measurable and less manageable realm of political and power dynamics” [7]. Health reform, particularly when aimed at enhancing universal health coverage, is a fundamentally political process with major collective action challenges, as it entails the redistribution of power and resources with inevitably winners and losers [8].

Consideration for the ‘enabling environment’, or political context in which technical health policy is conceived and implemented is therefore essential [9–11], especially given the situation of precarious statehood in Tajikistan. The leading research question of this article is therefore ‘what have been the main governance problems in the conception, development and implementation of the BBP and directly related health reforms?’ More generally, the Tajik case can help to answer questions such as: which institutional constraints can be identified to be standing in the way of health policy development and implementation? How do these mechanisms influence each other, and what lessons can be drawn from it?

This study aims to offer an insight into the interplay between the technical and political dimensions of health reform. The case of Tajikistan and the BBP reform provides an illustration of the way political dynamics in a fragile, post-conflict environment affect the design and implementation of a health financing reform. With a focus on the political economy dimensions of health governance, policy formulation and implementation the analysis is embedded in the wider debate on the drivers and spoilers of change in development policy, political economy of reform and generally the political dimensions of governance. Health governance in this study is defined as a process in which institutions, understood as the both formal and informal norms, rules and laws that shape the actions, and particularly the authorities, roles and accountabilities among societal actors in a health system [12–14]. These institutions influence the way a variety of state and non-state actors ‘make policy’ i.e. conceive of, formulate and implement it. The gap between agenda-setting and policy formulation on the one hand and policy implementation on the other is often striking, and has been the focus of a number of studies, albeit usually in more high income settings [15]. The former (agenda-setting and policy formulation) is here understood as the process in which various actors push their policy options and are ultimately adopted in formal laws, codes or rules, albeit in sometimes incoherent terms. The latter (implementation) can be defined as the way these codified practices are actually carried out by ‘street-level bureaucrats’ [16]. It is important to emphasise the heterogeneity of actors in this process, each influenced by different (formal and informal) institutions and networks with sometimes competing agendas as a result. This institutionalist perspective on governance and policymaking deviates from the ‘good governance’ or ‘best practice’ paradigm in which governance is viewed in a priori universally normative terms that are largely shaped by the experiences of legal-rational bureaucracies in high income settings, and tend to be more focused on the technical rather than the political dimensions of governance [7, 9, 17].

The debate on governance has diversified in recent years, and an increasing number of case studies from different sectors have enriched the body of evidence on the political economy of policy planning and implementation. However, this lens could be more applied to the field of global health to get a fuller understanding of policy processes and outcomes in the drive towards Universal Health Coverage [8, 18]. Particularly in Central Asia and Tajikistan, the political dimensions of health policy and governance remain underexplored. This research therefore contributes to the still limited body of evidence on the politics of health reform, and fills a gap in the literature on health governance in Central Asia.

The article is organized as follows. The next section describes the methods used to undertake the research in Tajikistan, which includes a discussion of the basic political economy analysis framework used for the combined process of data collection and analysis. The results section starts by describing the institutional and structural context, including some of the main characteristics of the Tajik political regime that shape the health system and its functioning. With this background the overall structure of the system and its associated challenges, including underfunding and fragmentation are outlined. The following section presents the findings on the structure-agency dynamics that form the main health governance constraints to the BBP reform in Tajikistan. The discussion attempts to synthesize the findings with the emerging theory on what the most pertinent constraints to effective health reform are. Lastly, the paper concludes.

## Methods

### Research design

The study design for this research is based on a case study design [19] allowing for in-depth exploration of a contemporary phenomenon in its real-life context, whereby the boundaries of the phenomenon are not nessarily evident. Case study approaches have been found to be particularly useful to understand and explain causal pathways in health policy developments and reforms [20].

### Data collection

Data for this study was collected through literature review as well as semi-structured and open, in-depth key informant interviews (KII). The review included grey literature retrieved through contacts in country and targeted internet searches from the websites of relevant organisations and institutions, as well as literature from a range of disciplines on Tajikistan’s political system, economy and health sector, identified through targeted internet searches and snowball sampling.

Purposive snowball sampling techniques [21] were used to identify key informants in the Tajik health governance context. The key informants were selected according to their expertise and level of involvement in the basic benefit package reforms, at both design and implementation levels. A total of 31 informants from governmental, bilateral, multilateral and non-governmental organizations, based in Dushanbe and various other parts of Tajikistan over the course of May 2010– December 2011 were conducted to gain insight into the governance, policy-making and reform context. Interviews with 23 local administrators, managers and health staff during the same time period in one BBP pilot district, as well as interviews with an additional five key administrators in two other BBP pilot districts were conducted for a detailed insight into the practical implementation of health policy. The purpose of the research was explained before each interview. References are not named in order to protect informants.

For data collection and analysis an exploratory approach was used following principles of grounded theory [22, 23], which in essence treats data collection and analysis as an interrelated process, as social phenomena are understood to be naturally dynamic and actors respond to changing conditions and the consequences of their actions and those of others. A topic guide was used for interviews that was focused on the main design and implementation challenges of the BBP reforms, key stakeholders in the health sector and their influence and accountability relations vis-a-vis each other. However, as data collection and analysis were undertaken in the same process, and an analysis of each interview was made before the next interview, the topic guide was updated and adjusted depending on the type of source and new insights gained during the process of data collection. Based on this approach, a number of themes on key governance constraints to health reform and implementation in Tajikistan emerged and hypotheses developed on their relations. These themes and hypotheses were in turn tested and adjusted during the course of data collection until sufficiently confirmed or ‘saturated’ [24].

### Data analysis

This study uses the basic features of political economy as a starting point for data analysis. Political economy analysis can broadly be defined as a set of methodologies based on economics applied to the analysis of political behaviour and institutions [25]. An important assumption underlying political economy analysis is that the governance context in which reforms of basic service sectors take place is shaped by formal and informal institutions, behavioural patterns, networks and agents which in turn influence the design and implementation of policies. In other words, the way policymakers and implementers act and perform is dependent on the, often heterogeneous, institutional environment in which they are embedded [26]. Within the given context individuals are assumed to act in their perceived best interest and form occasional coalitions with those who have similar interests [27] that may not be aligned with the goals of a given reform. As informal institutions shape behaviour and reproduce power, weak legal-rational bureaucratic structures can be pervaded, replaced and modified by more particularistic normative frameworks and relations, leading to what Eisenstadt termed neo-patrimonialism [28]. Although often criticized as being too broad of a concept without much explanatory power, neo-patrimonialism is here used to enable an understanding for the personalised type of political-bureaucratic constellation and authority that also characterizes the situation in most Central Asian countries, including Tajikistan [29]. A neo-patrimonial institutional setting is usually seen to be discouraging rigorous performance management or equitable public service delivery, and instead to be encouraging corruption and clientelism [9, 30, 31]. Political economy analysis is multifaceted with a wide array of approaches. However, common features include a focus on structures and institutions on the one hand, and agency dynamics, i.e. relevant actors, their interests, motivations and processes of cooperation and contestation on the other hand [27, 32]. These features formed the basic framework for the the first level of analysis in this research.

The following results section presents the main themes that emerged from the research in the following order. First of all, the relevant governance and health system structures, institutions, and actors are discussed. Secondly, the main structure-agency dynamics are presented that form the key governance constraints to the BBP reform and implementation.

## Results

### Governance and the health sector in Tajikistan: the institutional and health system context

#### Governance background Tajikistan

Partly as a consequence of the national state’s lack of resources to organize local systems, and partly as a legacy from the political settlement that ended the violent conflict in the 1990s informal power relations in Tajikistan have shaped the implementation of governmental policy [33–39]. In fact, since the period of Russian colonialism in the nineteenth century and even the Soviet period the direct influence of the state beyond the district level is limited, merely taking the shape of co-opted local elites [34, 40–42].

Political power is highly centralized in the position of President Emomalii Rahmon, while his domination of the political landscape depends on his ability to pacify a fragmented set of groups through the distribution of spoils and ‘virtual politics of peace’ [43]. In the face of the near-collapsed condition of the unitary state apparatus after the war, the power-building strategy of Rahmon, who has remained in power until this day, has been to either co-opt or neutralize political rivals through cronyism and repression [33, 44]. Ethno-regional identities and loyalties play a key role in this process [45]. Public services such as the security forces are not only attractive to work in because they provide access to a toll position, they also function to make people complicit in the system of rent-seeking and through that as an arena of acquiescence and political control [38, 44–46].

The relative, virtual peace that has prevailed in the country, save for sporadic violent outbreaks in the Gharm region, Gorno-Badakhshan and around Dushanbe, has as a result come at the detriment of legal-rational institution-building [43] and basic service delivery. Although corruption and cronyism certainly were not absent during Soviet times (e.g. as *blat*, as elaborated by Ledeneva [47]) a quarter of a century after its demise, the Soviet experience still stands in positive contrast to the current life conditions for a majority of the population^2^ [48]. Tajikistan remains the poorest of the former Soviet republics and that with the lowest Human Development Score. Its score trend over the period 1990–2015 in the Human Development Index suggests it is one of the countries with the most stagnant human development [49]. Moreover, positive economic growth since the end of the civil war is largely remittances-fuelled as they are estimated to make up 52% of the country’s GDP, the highest share of any country globally [50].

With a rent-seeking logic pervading the bureaucracy that is primarily aimed at short term patronage [37, 38, 44], non-productive sectors such as health face neglect and underfunding. Because of the intense personalization, and de facto patrimonialization of power, the Tajik state remains institutionally weak and operates under top-down rationale with limited bureaucratic capacity at the lower levels of government [51]. As a partial consequence, Tajik public function is characterized by little vertical accountability towards citizens, and top-down decision-making that is driven by political need and power dynamics at the top rather than evidence based-policymaking [1]. This authoritarian, personalised leadership with weak legal-rational institutions conforms closely to the dominant-discretionary ideal type, as developed by Levy [52], contrasting with more competitive and rule-of-law based political arrangements.

In the interaction with external donors, the Tajik government has become trained in adapting to the symbols and language of the international community [43] and has acquired an ability to instrumentalise assistance for its own goals [53] that has only been further refined over time. The interaction between this neo-patrimonial regime and a group of donors that have not closely harmonised their agendas and efforts has affected the state of the health system and the implementation of reforms, as this study suggests.

#### Health sector governance

The Tajik health system continues to formally resemble the Semashko organisational model put in place during Soviet times, with publicly owned, and -financed service providers wholly dominating the health sector. As originally devised the Tajik health system is still characterized by a frequent duplication of functions among agencies and administrative levels and a fragmented institutional setup [54]. Similar to the situation in other Central Asian countries, health facilities exist at the republican, oblast (regional), rayon (district) and jamoat (municipal) level and each different level of government performs similar and overlapping roles including revenue collection, provision of services, payment of salaries, maintenance of infrastructure, monitoring and enforcement [55]. Additionally, specialized health services for specific disease groups exist through vertical programmes, while some employers, including the Ministries of Defence and Internal Affairs run their own health services [4]. Private service provision is mainly limited to a few health providers in the capital on the other hand. Such a bureaucratically fragmented health system with duplication of functions not only leads to wastage of scarce resources, it also poses severe challenges in a context such as the one prevailing in Tajikistan where, as described above, the implementing capacity of the state is limited, especially at the local level [56].

The Tajik health sector continues to suffer from a lack of adequate public or risk-pooled funding as well as inequitable and inefficient financing practices. As Table 1 shows a comparison of Health Expenditure (HE) patterns in other low- and lower-middle-income post-Soviet countries suggests that public resources for health are comparatively limited in Tajikistan, have little priority in the government budget, and, probably as a result, out-of-pocket HE is comparatively high. As suggested by Xu et al. [57] this directly correlates with a high incidence of catastrophic and impoverishing HE by households. At 6.8% general government HE as a percentage of total government expenditure in Tajikistan is the third lowest of the WHO Europe region after Azerbaijan and Georgia.

**Table 1:** Health expenditure in Tajikistan and a selection of post-Soviet low- and and lower-middle-income countries

	Tajikistan	Kyrgyzstan	Uzbekistan	Moldova
Government HE [1] as % of total government expenditure	7	10	9	13
Government HE as % of total HE	28	45	53	46
OOP [2] expenditure as % of total HE	63	48	43	46

[1] Health Expenditure

[2] Out-of-pocket

All data from the WHO Global Health Expenditure Database, latest available data (2015)

Sources: Global Health Expenditure Database (WHO, latest available data)

As a non-productive sector, the Soviet health system already chronically suffered from the symptoms of a shortage economy: high shortage intensity, harder-than average budget constraints, and chronic under-fulfilment of supply, investment and output plans [58]. Financing of the healthcare system today remains largely input-based: although originally the infrastructure and resources for the health system were calculated upon basic population norms, the norms and subsequent line items were never adjusted [59] and had not been adjusted until the time of research. Since April 2014 Performance-Based Financing (PBF) has been piloted in Sughd oblast, followed by Khatlon oblast since early 2015. PBF complements and might partially replace the non-transparent input financing mechanism for health that is described in this study. However, due to its piloting nature in a limited part of the country it remained beyond the focus of this study.

In terms of system output, a pressure to ‘produce’ in Soviet times, based on quantity indicators, lead to a legacy of extensive coverage on the one hand but a surplus of narrow specialists and hospital infrastructure on the other hand. This has come at the expense of overall quality, efficiency and technological innovation; and PHC in particular [3, 4, 58, 60].

Following Tajikistan’s independence, a combination of a sudden stop of subsidies from Moscow, severe economic shock and civil war put a great strain to the state budget and subsequently the health system. As resources dwindled, existing weaknesses of the system worsened, and the quality of services deteriorated. Although informal out of pocket payments were certainly not absent in Semashko systems during the communist period, as studies in European countries suggest [61, 62] and reliable private HE data on Tajikistan from the 1980s and early 1990s is scarce, the large drop in public health spending,^3^ combined with evidence of big increases in out-of-pocket expenditure from studies in the Central Asian region suggests out of pocket payments, of which a substantial amount appears to be informal payments, came to increasingly fill this gap [1, 3, 58, 63–65]. A time-trend analysis of household surveys conducted in Tajikistan between 2005 and 2011 suggests the median amount of OOP, adjusted for inflation, doubled in that period [66].

To address the underfunding of the system, formalize informal payments and strengthen PHC, co-payment or user fee reforms have been initiated over the past decade. These include the co-payment regulations that are central to the BBP reform, which by 2011 had been piloted in eight districts^4^ with support from development partners, and the co-payment policy as outlined in governmental decree no. 600 (Decree 600), for which the Tajik government takes full responsibility. As analysed by Rechel and Khodjamurodov [2, 3]. The BBP guarantees a defined set of health services at no official charge for a limited number of population and patient categories.^5^ For all other care-seekers the BBP obliged to cover between 50 and 100% of ambulatory and diagnostic services costs depending on availability or not of referral from a PHC practitioner (50%) and place of residence (80% is charged to residents while 100% payment applies to those who seek care in rayons (districts) in which they are not a resident). In PHC consultations and treatment are provided free of charge apart from ambulatory services and diagnostic tests.

First introduced under Government resolution 237 (“on approval of the BBP for citizens of the Republic of Tajikistan and guidelines for the provision of medical and sanitary services by the state”) and implemented nationwide in 2005 the BBP was suspended within months after heavy criticism from development partners and healthcare professionals. The criticism centred around the lack of accompanying financing mechanisms to rationalise and increase funding for PHC, the unpreparedness of all affected by the implementation of the reform including lack of capacity-building of health workers and administrators to implement the provisions of the reform and the complexity and lack of standardisation of co-payment categories and rates (KII and [2]). Following extensive consultations between the Ministry of Health (MoH) and development partners, a revised BBP was introduced in pilot districts in 2007.

The new payment structure aims to realign the financial incentives for patients to increase the use of PHC facilities in their own jurisdiction and reduce incentives to use hospital level care as the entry point to the health system. The introduction of exemption categories has the goal of preserving and even enhancing affordability of health services for certain vulnerable groups. The introduction of the BBP has been accompanied by two other relevant reforms. Under governmental decree No. 665 that was passed in 2009, district health departments (RaZdrav or GorZdrav) were established, formally shifting coordination of health service delivery at this level away from the previously responsible chief physician of district hospitals. In some districts in which the district’s capital authorities are tasked with the coordination of health care services this committee is usually referred to as GorZdrav. Its purpose is however identical and the body will therefore be referred to as RaZdrav in the rest of the article.

Governmental decree 600, passed in 2008, introduced a separate set of user fees for 1200 different services, with much similarity to the failed 2005 BBP policy. The fee levels and categories were not synchronised with the newly revised co-payment regulations under the BBP and no fee exemption mechanism was in place, the levels and rates were not transparent for patients and were too complicated to manage without risks of supplier-induced demand. After intensive discussions, the MoH, together with USAID’s ZdravPlus II project, worked to simplify the co-payment structure and started piloting it in 13 hospitals around the country [67]. The co-payment structure and regulations on the use of user fee revenue was however still not synchronised with that of BBP at the time of research. Given the limited scope of Decree 600 at the time of research this article is focused on the BBP and its related changes to the health governance structure, i.e. the introduction of a PHC manager and the RaZdrav committee as introduced under Decree 665.

#### Main formal actors in the system

Apart from the MoH, as the formal steward of the health system, the most influential actors in the health system in terms of political power at national level are the Ministry of Finance, the president and his shadow administration, made up of advisors who remain beyond legislative control as Abduallaev already found [68], and bilateral and multilateral donors that have funding leverage, but whose efforts have since the end of the civil war not been strongly coordinated or harmonised [1, 2, 69, 70]. The main international donors have been represented in a donor coordination council that has officially been chaired by President Rahmon. As will be elaborated later, the council has not functioned as a body to actively coordinate or collaborate on incorporating lessons learned or using common guidelines in piloting the BBP either between donors or with the government. Rather, it remained a body that merely served the purpose of information sharing [1]. At the district level, formally the main actors are the District Hospital Director, the district health committee *RaZdrav*, the PHC manager, and the district’s financial department (*GorFin*). In the BBP pilot districts different development agencies, through their relevant health programme staff, assist in the implementation of BBP and related reforms.

### BBP’s key governance constraints: an exploration of structure-agency dynamics

The next section presents the main factors impeding the policy development and implementation of the BBP and related reforms at different interconnected levels in Tajikistan that emerged as themes from the field research findings. It attempts to highlight the interplay between the institutional/structure and agency dimensions of health policymaking and implementation as exemplified by the case of the BBP and associated changes in district health management.

#### Parallel and competing central government mandates, policy incoherence and regulatory fragmentation

A leading overarching concern on the BBP implementation, coming out of most KII, that affects all other governance constraints is the lack of adequately defined and understood policies, rules and mandates. A lack of clarity on which national government actor is primarily responsible for different decision-making and implementation processes, leads to policy incoherence, duplication and fragmentation of responsibilities at governmental level [71]. This is exemplified by the existence of parallel and competing government structures with unclear attributions and mandates. The roles of ministries, such as those for health and finance that fall under the prime minister’s office are often duplicated by sector heads and specialists under the President’s executive administration, whose authority is beyond legislative control. Most of these actors are represented on the coordination council that has existed since 2011, in which government actors and donors meet to discuss health initiatives, while their exact responsibilities and powers remain unclear. The lack of collaboration in the relationship between these segments of the government became evident during discussions on reform implying a purchaser provider split. Despite this being an agreed-upon goal in the national health strategy to which the MoH subscribed, the Ministry of Finance was strongly opposed as it meant devolving its purchaser-role to the regional level. Only after a donor appeal to the Presidential administration the Ministry of Finance ultimately agreed (KII).

#### Overcharging and informal payments

Policy incoherence has had a marked influence on the extent to which implementation of BBP payment schedules and exemption guidelines is non-arbitrary, leading to an increased opportunity space for actors to use their public office for private gain (KII). Combined with the general scarcity of resources this fragmentation and vaguely, sometimes contradictorily formulated rules and procedures were perceived to facilitate the rent-seeking behaviour of staff in key positions, expressed in informal payments for patients, and the ensuing power-play between them over their mandates (KII). The documented variation across facilities and rayons in which co-payments are charged under BBP supported by the SDC-funded Project Sino^6^ [66, 72, 73] indeed suggests erratic enforcement of BBP guidelines, possibly facilitated by a lack of awareness on behalf of both patients and providers. Exemption and co-payment categories have been reformulated in a short space of time, and have been piloted by different donors with their own variations on the programme leading to additional confusion for health staff and patients. As documented this erratic implementation of BBP payment guidelines in practice means there is a tendency for excessive charging, including 100% fees for district residents, who are entitled to reduced rates [73], and payment for nominally free PHC services [74]. The general situation of underfunding in the health system has not helped to reduce informal payments substantially. Rather, the intense financial constraints serve as a powerful incentive for the responsible administrators to acquire income through a system of upward channelling of proceeds from informal payments at health facility level (KII).

#### Weak budgeting practices

An important factor intensifying the fragmentation is the weak link between budgeting and policymaking at the republican level government of Tajikistan. KII with respondents from development agencies, the ministries of finance and health indicated that this regularly resulted in the development of strategically formulated policies for which no adequate or sustainable sources of funding existed. The lack of an implementation budget for the BBP and the lack of an independent budget for the RaZdrav to conduct monitoring and regulatory work are examples of this policymaking – budgeting rift. This is aggravated by the Ministry of Health’s lack of budgetary autonomy as the vast majority of funds for healthcare is directly channelled from the Ministry of Finance to local levels of government, as explained below. The government’s adaptability to the language of the donor community and the donors’ pressure to execute funding often led to these gaps being compensated for with external funds, which would usually be committed only ad-hoc or for a few years (KII). Although weak technical and institutional capacity at the MoH plays an important role [2], the practice can also be sustained by continuing donor commitment without large costs for the government following the principles of moral hazard. In the absence of a functioning formal mechanism of budget allocation, bargaining power towards the political-administrative capital Dushanbe has become and remains an important determinant in budgeting (KII), resulting in inequities between rayons. Consistent with the political regime analysis discussed above, KII with financial and health administrators from three different districts confirm previous observations [1, 3], that although local budget requests are sent to Dushanbe, decisions on budgetary allocations are ultimately taken following a non-transparent logic at the Ministry of Finance. The MoH is effectively sidelined in this process, with rayons in practice bargaining for their health funding directly with the Ministry of Finance (KII).

#### Little donor harmonisation

The behaviour of development agencies in the BBP reforms has further contributed to policy incoherence and regulatory fragmentation. Objectives, perspectives and modes of operation and evaluation have varied considerably between donors in Tajikistan. Until the establishment of the Health Coordination Council in 2011 there had not been a formal body for aid coordination in the health sector between donors and the government, as donor-government contact mainly took place on an ad-hoc or bilateral basis (KII). Aid coordination has in practice mainly implied the sharing of information on aid activities under the auspicies of the Ministry of Finance [6]. Development partners, of which the most important actors have been SDC, USAID, DfID, WHO, EU, the WB and ADB have often emphasized different elements of health reform and some ran only short-term pilots, adding to the lack of clarity for providers and patients on co-payment policies (KII). Although the National Health Strategies have helped to formulate a direction, which could function as a basis for some level of accountability, an agreed timeline for piloting reform initiatives and scale-up or a systematic effort at monitoring and evaluation for these pilots has never existed.

#### Weak accountability to citizens

As [2] have outlined, national health governance and in particular the development of the BBP reforms has been characterized by a lack of participation of non-state actors or lower levels of government. This is matched by the lack of a strong legislative at district level of government. KIIs suggested decision-making at rayon level, where health reforms are implemented, is dominated by the district chairman, or *rayon rais*, who is appointed by the president’s office, and in turn appoints municipal mayors. Although an assembly of deputies exists in every rayon, it was considered to hold merely ‘consultative status’ by local government officials (KII). Furthermore, the President’s People’s Democratic Party has held absolute majorities in parliaments since the end of the civil war, and according to human rights watchdog Freedom House political rights have been severely curtailed by the government, “sustaining a campaign of repression against opposition, dissent, and criticism” [75]. The absence of competitive electoral politics is a possible explanation for the lack of pork-barrel politics observed. Rather than such a prototypical clientelistic setup, where benefits are delivered to constituencies of citizens in exchange for political support, a system of pervasive bottom-up rent extraction in the Tajik health sector was a widespread perception surfacing in KII. This is in line with the fact that, despite the direct appointment of cronies from Dangara and Kulyob, the president’s home base, to powerful government positions, the districts themselves remain poor and badly serviced [37]. Similarly, health facilities in Tursunzade, one of the BBP pilot districts, is just as poorly equipped, with a patchy supply of electricity and water, despite its economic importance for the political centre, as that in the rest of the country (KII and personal observation).

## Discussion

This study has provided an insight into the relevance of the political-institutional context to health reforms by analysing the governance constraints to the BBP reforms in Tajikistan. The findings from desk research and KII suggest that little donor harmonisation, policy incoherence, parallel and competing central government mandates, and regulatory fragmentation, stand out as dominant drivers of most other constraints to effective design and implementation of the BBP and associated health reforms in Tajikistan: overcharging and informal payments, a weak link between budgeting and policymaking, a practice of non-transparent budget bargaining instead of a rationalisation of health expenditure, and weak accountability to citizens. Beyond identifying these governance constraints per se the findings serve to illustrate the complex and interlinking structure-agency dynamics that impact health sector reforms in neo-patrimonial settings. In this section, the findings are synthesised with the existing evidence from other cases to draw conclusions on the institutional constraints to effective service delivery reform and their interlinkages, and provide recommendations.

The interplay between institutional/structural factors and agency is particularly highlighted in the way that policy incoherence and regulatory fragmentation around health financing and management was found to be largely a consequence of the combination of uncoordinated donor pressures for health financing and management changes, and the existence of governance actors with unclear, parallel and competing mandates at the central level. The role of aid in health systems strengthening in particular and public sector reform in general has been widely discussed (e.g. [76–80]). In line with the wider literature the findings from this study illustrate how a lack of donor harmonisation can create and exacerbate fragmentation of the health system. The finding that external pressure for health reform from different development actors without central prioritisation or sufficient engagement with implementing actors nor a realistic timeframe has impeded a coherent introduction of the BBP, mirrors health reform processes in other fragile and post-conflict settings [81, 82]. Different waves of piloting the BBP concept, executed by different development agencies have produced a landscape of incoherent mandates for new positions and guidelines for fee-charging. Harmonising the technical and political objectives behind development cooperation carries an inherent challenge [7]. The incentives that different development agencies face with their own programming cycles, policy agendas, domestic constituencies and deliverables are not always conducive to donor harmonisation [78, 83, 84]. Furthermore, as a study of health policymaking in Cambodia and Pakistan demonstrates, power between donors and government actors is asymmetrical and exercised not only through financial resources, but also technical expertise and evidence-generating capacity, thereby setting the agenda for policy reform [85]. In a fragmented aid landscape this highly complicates the possibility of keeping health financing policies coherent. What this study additionally shows, is that support for health reform that is not sufficiently coherent, harmonised and focused on the long term not only leads to moral hazard, but affects the power balance between governance actors (inter-departmentally and between ministries and the presidential cabinet), echoing findings from Uganda [79].

The findings from this study suggests that policy incoherence and unclear mandates, in combination with deep underfunding creates an opportunity case for the widely reported phenomenon of bottom-up financing of health providers and authorities, partly expressed in the recorded high degree of overcharging of user fees and informal payments. This corresponds to rent-seeking phenomena in other neo-patrimonial settings, such as the ‘ascendant financing’ mechanism (referred to as ‘the pump’) in Democratic Republic of Congo [86, 87], and the low adherence to fee exemption rules in Burkina Faso [88]. The bottom-up financing mechanism may suggest that management positions in the health sector can function as toll positions from where rent can be accrued, similar to rent-seeking patterns in the wider bureaucracy and land governance in Tajikistan [33, 46, 89, 90]. In other words, policy incoherence, the lack of clearly defined mandates and lack of resources to carry out basic tasks of health provision, regulation and oversight at the local level are not only features of neo-patrimonialism but also create the conditions for patrimonial features of governance to penetrate legal-rational bureaucracies. This highlights the dilemmas that aid can perpetuate or entrench power relations and control of resources, as Nakaya found in the phase of early recovery Tajikistan [37] and that the ideal of national ownership can in practice imply control by authoritarian elites in closed political environments, as found in Rwanda [91]. As North et al. (2006) observe, rent-seeking is inherent to all political systems but as rent-seeking and limiting of privilege increases, the economy generally shrinks and with it the possibility of broad tax-based developmental programmes. In recent years, the negative correlation between neo-patrimonialism and development has been nuanced with analyses of the varying performance of neo-patrimonial settings depending on the extent and type of rent-seeking behaviour [92–94]. Rent management through personalised top-down patronage can work out in both predatory and developmentalist ways [52]. Rather, what appear to be decisive factors is whether these rents are accrued from productive or unproductive sectors, centralised, and geared to long or short term interests [95]. What sets Tajikistan apart from the more developmentalist cases (e.g. Rwanda, Ethiopia and China) is the combination of weak bureaucratic capacity with short term, fragmented developmental planning and management by the elite, as findings from this study suggest. Quick overturn of staff at the central level, often for the purpose of political neutralization [33, 37], further contributes to the loss of institutional memory, strategic vision and commitment to carry out previously-agreed reforms. Neo-patrimonialism and associated patterns of rent-seeking can thus be a cause and a consequence of policy incoherence.

Lastly, accountability from civil society organisations and citizens is often seen as crucial to strengthen more equitable and responsive health services [96]. However this study has posited that a lack of bottom-up bargaining power or limited ability to demand accountability on performance is a central feature of the political arrangement in Tajikistan, where patronage finds expression in appointments of cronies to key positions in public service to accrue rent, rather than clientelist relations between ‘big men’ and their constituents [97]. The findings suggests that in this context opportunities for citizen involvement in policymaking are very limited in general. This speaks to the findings from three other post-Soviet republics that a hostile political and economic climate limits the potential for civil society advocacy [98]. In such a context fear for personal safety, losing out on contracts or other types of exclusion is a dominant disincentive for civil society engagement and government criticism. In terms of policy development and implementation it risks marginalizing the voice of underrepresented and vulnerable professional or patient groups but is also an impediment to understanding local public health needs.

As with any policy reform analysis this study has been subject to limitations and its results are highly time-bound to the period of field research. Policy details have changed and will continue to change as new reforms are piloted, terminated or altered. Some of the limitations to this research are inherent to its approach and focus. Exploring the ‘murkier realm of politics’ in a neo-patrimonial, closed and authoritarian political setting is delicate as it touches upon often conflicting interests and therefore requires provisions in the presentation of results to protect informants. Further research in this area is therefore warranted. This includes a deeper exploration of de facto health financing arrangements, such as the health funding allocation mechanisms, and the dynamics around informal payments and their perceived channelling upwards, but also more in-depth research into accountability relations between providers, regulators and citizens at the local level of implementation.

## Conclusions

Studying the political and institutional constraints to health reform is key to better understand the incentives and motivations that further or block improvements in public health. This study raises a number of previously under -researched health policy development and implementation challenges in Tajikistan. In doing so it not only contributes to the small body of literature on public sector reform in Central Asia and Tajikistan in particular, but also to the growing literature on the political constraints to aid and health reform in general. Based on the example of the BBP reform this study has found that health reform in Tajikistan suffers from a combination of policy incoherence, parallel and competing central government mandates, and regulatory fragmentation. This finds an expression in weak budgeting practices and overcharging of user fees. Rent-seeking patterns were widely reported to play a role in this, and poor coordination between external development actors has added to these challenges. The article points to the importance of considering the political-institutional context in which reforms and indeed donor interventions take place. The findings raise pertinent questions on the unintended consequences of non-harmonised piloting of health reforms, and the interaction of health financing interventions with entrenched power relations. These findings can encourage reflection on the relevance of contextualising health programmes and addressing policy incoherence with long horizon planning as a priority.

## Footnotes

1 This health system model is named after Nikolai Semashko (1874–1949), the Soviet Union’s first ‘People’s Commissar of Public Health’ (Minister of Health) who laid the foundations of the Soviet health system, which also served as a blueprint for health systems in most socialist economies. Common characteristics are the publicly funded financing model, with no formal charges at the point of service delivery; public ownership and management of health facilities in cascading levels of specialization from municipal, district, region and state level; and relatively high levels of specialization, human resources for health and hospital infrastructure.

2 In a Gallup poll undertaken in Tajikistan in 2013 only 27% of respondents replied they think the breakup of the Soviet Union benefited the country, and 52% said it harmed the country. This confirms personal observation from the vast majority of respondents who reminisce positively about life in Soviet times.

3 By 1994 real per capita public health expenditure had dropped to 46% of its 1990 levels [63]

4 The first four plilot rayons from 2007 onwards were: Dangara, Spitamen, Tursunzade, Rasht. In 2009 BBP piloting was extended to the districts of Shahrinav, Varzob, Sarband, Nurek town.

5 Twelve social groups (such as veterans, elderly, infants) and fifteen disease groups (including TB, HIV/AIDS, leprosy, malaria) are exempted from these co-payments

6 These were the districts of Tursunzade, Shakhrinav, Dangara, and Varzob at the time of research.
